# Therapeutic Potential of Oxytocin in Atherosclerotic Cardiovascular Disease: Mechanisms and Signaling Pathways

**DOI:** 10.3389/fnins.2019.00454

**Published:** 2019-05-21

**Authors:** Ping Wang, Stephani C. Wang, Haipeng Yang, Chunmei Lv, Shuwei Jia, Xiaoyu Liu, Xiaoran Wang, Dexin Meng, Danian Qin, Hui Zhu, Yu-Feng Wang

**Affiliations:** ^1^Department of Genetics, School of Basic Medical Sciences, Harbin Medical University, Harbin, China; ^2^Department of Medicine, Albany Medical Center, Albany, NY, United States; ^3^Department of Pediatrics, The Forth Affiliated Hospital, Harbin Medical University, Harbin, China; ^4^Department of Physiology, School of Basic Medical Sciences, Harbin Medical University, Harbin, China; ^5^Department of Physiology, Jiamusi University, Jiamusi, China; ^6^Department of Physiology, Shantou University of Medical College, Shantou, China

**Keywords:** atherosclerosis, coronary artery disease, etiology, oxytocin, signaling pathways

## Abstract

Coronary artery disease (CAD) is a major cardiovascular disease responsible for high morbidity and mortality worldwide. The major pathophysiological basis of CAD is atherosclerosis in association with varieties of immunometabolic disorders that can suppress oxytocin (OT) receptor (OTR) signaling in the cardiovascular system (CVS). By contrast, OT not only maintains cardiovascular integrity but also has the potential to suppress and even reverse atherosclerotic alterations and CAD. These protective effects of OT are associated with its protection of the heart and blood vessels from immunometabolic injuries and the resultant inflammation and apoptosis through both peripheral and central approaches. As a result, OT can decelerate the progression of atherosclerosis and facilitate the recovery of CVS from these injuries. At the cellular level, the protective effect of OT on CVS involves a broad array of OTR signaling events. These signals mainly belong to the reperfusion injury salvage kinase pathway that is composed of phosphatidylinositol 3-kinase-Akt-endothelial nitric oxide synthase cascades and extracellular signal-regulated protein kinase 1/2. Additionally, AMP-activated protein kinase, Ca^2+^/calmodulin-dependent protein kinase signaling and many others are also implicated in OTR signaling in the CVS protection. These signaling events interact coordinately at many levels to suppress the production of inflammatory cytokines and the activation of apoptotic pathways. A particular target of these signaling events is endoplasmic reticulum (ER) stress and mitochondrial oxidative stress that interact through mitochondria-associated ER membrane. In contrast to these protective effects and machineries, rare but serious cardiovascular disturbances were also reported in labor induction and animal studies including hypotension, reflexive tachycardia, coronary spasm or thrombosis and allergy. Here, we review our current understanding of the protective effect of OT against varieties of atherosclerotic etiologies as well as the approaches and underlying mechanisms of these effects. Moreover, potential cardiovascular disturbances following OT application are also discussed to avoid unwanted effects in clinical trials of OT usages.

## Introduction

Cardiovascular disease (CVD) is responsible for both high morbidity and mortality worldwide with coronary artery disease (CAD) being the leading cause of death (45.1%) (Cassar et al., 2009). In the United States, the prevalence of CVD comprising chronic heart disease, heart failure, and hypertension in adults (≥20 years of age) was 121.5 million in 2016 and increases with advancing age in both males and females (Benjamin et al., 2019). The major pathological basis of CAD is atherosclerosis that affects various components of the cardiovascular system (CVS), particularly the coronary artery. Over the past decades, identifying and reversing the pathogenesis of CAD and preventing myocardial infarction (MI) remain a major challenge for clinical management of CAD (Koton et al., 2014).

Oxytocin (OT), a nonapeptide synthesized in hypothalamic magnocellular neuroendocrine cells in the supraoptic and paraventricular nuclei (SON and PVN) (Yang et al., 2013; Johnson and Young, 2017), has emerged as an efficient cardioprotective agent in animal studies (Jankowski et al., 2016). However, due to the lack of deep knowledge of its involvement in the pathogenesis of atherosclerosis, therapeutic potential of OT in treating CAD is largely unexplored in clinical studies. In this review, we summarize our current understanding of the pathogenetic involvement of OT in atherosclerosis, the cellular/molecular mechanisms underlying OT suppression of atherosclerosis development and current challenges in clinical trials of OT.

## Atherosclerosis and Cardioprotective Effect of OT

Atherosclerosis can occur in the coronary artery, renal and cerebral circulation, and peripheral and mesenteric vasculature, largely due to metabolic disorders and immunological injuries (Rahman and Woollard, 2017). CVDs are often accompanied by disruption of OT/OT receptor (OTR) signaling. For instance, particulate matter 2.5 exposure resulted in global adult cardiac dysfunction (Tanwar et al., 2017) while reduced OTR mRNA expression (Iobst et al., 2019). Moreover, endoplasmic reticulum (ER) stress, a common cause of cardiovascular disorders (Wang S.C. et al., 2018), significantly reduced the levels of OT mRNA (Morishita et al., 2011); in ischemia/reperfusion (I/R) injury in C57B6 mice, OTR expression decreased in the heart by 40% (Indrambarya et al., 2009). By contrast, OT can exert cardiovascular protective function through suppressing the development of atherosclerosis and repairing the injured heart following myocardial infarction as shown in mice (Plante et al., 2015).

### Atherosclerosis and Its Etiology

Cardiovascular health is largely determined by mechanisms of vascular endothelial defenses. This defense involves oxygen utilization, tension on the wall and flow resistance, local regulation of vascular tone and contractility, control of inflammatory cell adhesion, and anti-thrombotic nature of the endothelial surfaces. These factors together support normal circulatory function and its adaptive response to adverse environmental challenges, disturbance of which can predispose to atherosclerosis (Kim et al., 2013; Lee et al., 2013). Factors leading to atherosclerosis include consumption of high-fat and cholesterol diet (Marir et al., 2013), dyslipidemia (Sanin et al., 2017), diabetes (Lehrke and Marx, 2017), chronic inflammation (Lee et al., 2018), genetic risk (Whayne, and Saha, 2019), lack of exercise (Yang J. et al., 2017), hypertension (Hurtubise et al., 2016), social stress (Meng et al., 2019), smoking and other unhealthy life-styles or environmental factors (Niemann et al., 2017). As shown in a cohort study in Mexico, the coronary risk factors observed were dyslipidemia (100%), hypertension (86%), obesity/overweight (75%), metabolic syndrome (71%), smoking (68%), and diabetes (58%) (Rettori et al., 2014). Moreover, coronary artery spasm and embolism could be evoked by emotional or physical stress due to increased sympathetic output (Kc and Dick, 2010; Mori et al., 2012). Thus, immunometabolic disorders and abnormal cerebral-cardiovascular communication are the major etiology of CAD in association with atherosclerosis.

In general, the development of atherosclerosis begins with the attachment and invasion of leukocytes to the endothelium of the artery under the drive of oxidized lipoprotein particles within the wall or injuries of epithelial cells. The ensuing inflammation causes adhesion of platelets, leading to formation of atheromatous plaques in the arterial tunica intima. This process involves precipitation and oxidization of cholesterol released from circulating low density lipoprotein, inflammation-stimulated proliferation and migration of smooth muscle from the tunica media into the intima, followed by the formation of fibrous capsule and calcification of the arterial walls. As the plaques grow, wall thickening and narrowing can affect any arteries, particularly the coronary artery, resulting in a shortage of blood and oxygen delivery to various tissues. Pieces of plaque can also break off, and lead to MI, stroke, or heart failure if left untreated (Crea and Libby, 2017).

In the pathogenesis of atherosclerosis, different etiologies work through different mechanisms. For example, high blood cholesterol and fat can deposit in the wall of arteries, which reduces the flexibility of blood vessels, stimulates inflammation and restricts, even blocks blood circulation to the heart and other organs (Garrott et al., 2017). Hypertension can damage the endothelium of blood vessels in heart, increase shear and tear forces on vessel walls that change cell osmotic stability (Jiao et al., 2017) and fasten lipid deposition in arteries (Lawes et al., 2008). Cigarette smoking can damage the endothelium of arteries, stimulate inflammation (Hussien and Mousa, 2016), increase blood pressure (BP) and cause cardiac hypertrophy (Guasch and Gilsanz, 2016). People with diabetes have a much higher incidence of CAD because of dyslipidemia and metabolic disorders, particularly the formation of glycated proteins that cause inflammation, stiffening arteries, trapping other macromolecules, and disrupting enzyme activities, hormone regulation, immune function and activities of dendritic cells (Price and Knight, 2007). Furthermore, lack of social support or mental stress is etiologically related to coronary artery lesion through sympathetic-adrenomedullary influences on platelet function, heart rate, and BP in the initial endothelial injury, and the hypothalamic-pituitary-adrenocortical (HPA) axis that is involved in smooth muscle cell proliferation during progression of vascular lesion (Dupont et al., 2014). With increase in age, arteries become less elastic and are more susceptible to plaque buildup (Rahman and Woollard, 2017; Sanin et al., 2017). Correspondingly, prevention of CAD involves changing lifestyle to limit the amount of fat and cholesterol intake and increase their utilization through exercise. In the treatment, cholesterol-lowering medication statins, antiplatelet and anticoagulants, β-blockers, calcium channel blockers, diuretics, or angiotensin converting enzyme inhibitors can be recommended. In some cases, interventions may be necessary such as percutaneous coronary intervention, bypass surgery, thrombolytic therapy, angioplasty, and endarterectomy (Lee et al., 2013; Lindahl et al., 2017). These measures could slow down injuries to the arteries but with various side effects and complications. Thus, agents targeting multiple aspects of atherosclerotic pathogenesis or CAD but having minimal side-effects are especially critical, and OT is one potential agent with such properties.

### Cardiovascular Protective Effect of OT

Oxytocin has multiple cardiovascular protective functions, which are achieved through both central and peripheral approaches (Viero et al., 2010). OT terminals were found on large intracerebral (Zimmerman et al., 1984) and other large blood vessels (Jankowski et al., 2000) as well as neural structures regulating cardiac activity (Yang et al., 2013). OTR was localized in microvessels expressing CD31 marker and co-localized with endothelial NO synthase (eNOS) (Jankowski et al., 2010b). OTR mRNA was found in the vena cava, pulmonary veins, and pulmonary artery with lower levels in the aorta in the rats (Jankowski et al., 2000; Wsol et al., 2016). Thus, OT may modulate cardiac activity by activating OTRs on CVS.

#### OT and Ischemic Cardiomyopathy

Ischemic cardiomyopathy involves poor perfusion and oxygenation to the myocardium, mainly due to CAD. OTR signaling is closely associated with the development of CAD and its complications. As previously reviewed (Li et al., 2017), plasma OT levels or hypothalamic OT neuronal activities were significantly increased at the early stage of sepsis, advanced cancer patients, adjuvant arthritis and pancreatic injury, which in turn changed the activity of immune system to initiate immune defense, thereby playing the role of immune surveillance. In CVS, OT plasma levels and the activity of the intracardiac OT system significantly increased at 4 weeks after MI in the rats survived from the coronary artery ligation (Ciosek and Drobnik, 2012); post-infarction heart failure was associated with an increased activity of the intracardiac OTergic system (Wsol et al., 2016). Moreover, MI activates parvocellular OT neurons projecting to the rostral ventral lateral medulla (Roy et al., 2018). Thus, changes in the activity of the OT-secreting system in the brain and the heart can be a biomarker of immune disturbance in CVD while exerting the function of adaptive cardioprotection (Jankowski et al., 2010a).

The cardioprotective effect of OT has been proven by many experimental observations. OT administration significantly inhibited myocardial injury in rats (Moghimian et al., 2012; Polshekan et al., 2016); however, blocking OTRs with atosiban increased infarct size and levels of creatine kinase MB isoenzyme and lactate dehydrogenase (Moghimian et al., 2012; Houshmand et al., 2015). In rabbits with I/R of the left coronary artery, OT pretreatment significantly decreased infarct size and yielded antiarrhythmic effects including ventricular tachycardia and fibrillation (Faghihi et al., 2012); however, atosiban abolished the beneficial effects of this ischemic preconditioning of OT (Das and Sarkar, 2012). Lastly, in the post-ischemia repair of a rabbit model, post-infarction group treated with OT had reduced infarct size and improved left ventricular function by enhancing anti-fibrotic and angiogenic effects via activating OTRs (Kobayashi et al., 2009). Importantly, the cardioprotective effect of OT can be achieved in doses of 8 × 10^-12^ to 2 × 10^-11^M as shown in rat I/R heart (Anvari et al., 2012), a physiological level in the plasma (Hirst et al., 1991), indicating that the protective role can be physiological.

The protective effect of OT is first attributable to its negative chronotropic and inotropic roles in cardiac activity following the activation of cardiac OTR (Costa E Sousa et al., 2005) in association with the release of protective atrial natriuretic peptide (ANP) and nitric oxide (NO) (Houshmand et al., 2015) and increase in parasympathetic output (Sun et al., 2015), decrease in the activity of renin-angiotensin-aldosterone system (Nielsen et al., 1997) and reduction of sympathetic outflow (Olszewski et al., 2010). In addition, OT also decreased cardiac preload and afterload through its diuresis and natriuretic effect (Nielsen et al., 1997). As a result, OT can reduce oxygen consumption while increasing the cardiac output during I/R injury.

#### Vascular Protection

Oxytocin is synthesized and released in the heart and vasculature that express OTRs, and is important in normal homeostatic regulation of cardiac and vascular systems (Japundzic-Zigon, 2013). It is well-known that OT preconditioning increased expression of genes associated with angiogenic, antiapoptotic, and cardiac antiremodeling properties (Noiseux et al., 2012). OT promoted angiogenic behaviors of human umbilical vein endothelial cells through activating OTRs (Cattaneo et al., 2008) by increasing hypoxia-inducible factor-1α mRNA and protein expression (Zhu et al., 2017). In rats of sinoaortic denervation, intravenous application of OT induced an enhanced initial pressor effect with much reduced reflex bradycardia and fall in cardiac output. A larger and more prolonged delayed fall in mean arterial pressure was apparent with both OT and its specific agonist [Thr4,Gly7]OT (Busnelli et al., 2013) although supraphysiological doses of OT caused transient pressor reaction by activating vasopressin (VP) receptors (Petty et al., 1985). Moreover, OT could antagonize the pressor effect of VP through reflexively activating cholinergic neurons (Mukaddam-Daher et al., 2001). These effects allow OT to reduce the pre- and after-load of CVS.

The protective effect of OT is closely associated with its suppression of immunological disorders. In rat heart, angiogenic and antiapototic effects of OT were mediated by upregulating vascular endothelial growth factor (VEGF) and prosurvival B-cell lymphoma-2 protein (Kobayashi et al., 2009), with decreasing apoptosis caused by neutrophils (Al-Amran and Shahkolahi, 2013). Moreover, incubation of cells at physiological levels of OT significantly decreased basal and stimulated NADPH-dependent superoxide activity in vascular cells, monocytes, and macrophages that express OTR protein and mRNA. OT can decrease NADPH-dependent superoxide production and pro-inflammatory cytokine release from vascular endothelial cells and macrophages, and thus, inhibit inflammation and atherosclerotic lesion development (Wang P. et al., 2015). In Watanabe Heritable Hyperlipidemic rabbit, a model of dyslipidemia and atherosclerosis, chronic OT-treatment significantly reduced plasma C-reactive protein levels, atherosclerosis formation in the thoracic aorta and cytokine gene expression in visceral adipose tissues; however, body weight, serum lipids, plasma/urinary measures of oxidative stress, plasma cortisol, or urinary catecholamines did not change (Szeto et al., 2013). Thus, attenuating vascular oxidative stress and inflammation are important mechanisms for OT to antagonize the pathogenesis of atherosclerosis.

#### Cardioprotection in Post-menopausal Women

Coronary artery disease is generally considered the pathology of aging and gender with strong correlation with the activity of OT neurons. Between age 45 to 65, approximately 10% women developed CAD, while the incidence increased to 33% after age 65 (Benjamin et al., 2019). Correspondingly, heart disease was the leading cause of death for women in the United States, killing 289,758 women in 2013 (Xu et al., 2016). CAD-associated hypertension increased dramatically in women after menopause due to reduction in ovarian hormone in older women (McLeod et al., 2010), which could impair baroreflex and autonomic balance by negatively impacting OTergic drive and OT levels in pre-autonomic neurons in rats (De Melo et al., 2016). Declines in OT and OTRs were related to aging-associated acceleration of inflammation and oxidative injuries in the CVS, particularly after menopause (Light et al., 2005). Consistently, plasma OT level experienced a threefold decline in aged mice compared with young, and this decline was accompanied by similar decrease in levels of OTRs in muscle stem cells (Elabd et al., 2014). Exogenous estrogen application was found to increase OT secretion in both rodents (Quinones-Jenab et al., 1997) and women (Chiodera et al., 1991), and increase in OTR mRNA expression in mouse brain (Quinones-Jenab et al., 1997) as well as intracardiac OTR signaling (Jankowski et al., 2010b). The pro-synthetic function of estrogen in OT expression may also explain the lower CAD prevalence among women before menopause. Hence, OT has special potential in treating female patients with CAD.

## Mechanisms Underlying the Protective Effects of OT

**FIGURE 1:**
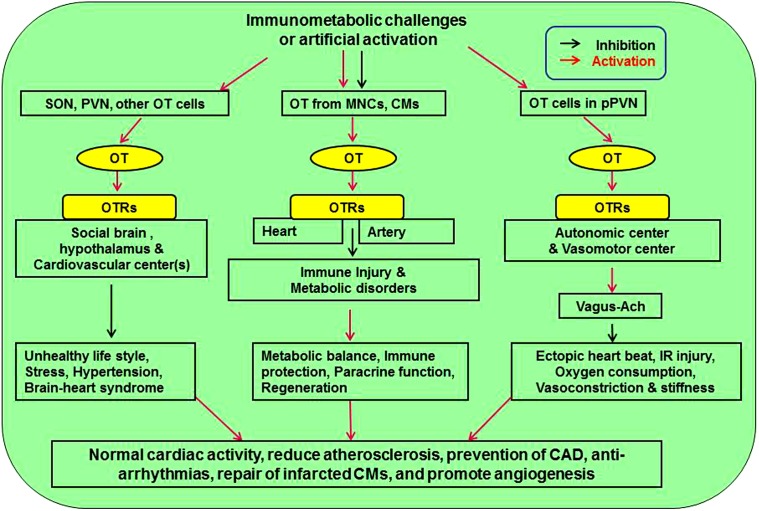
Approaches mediating cardiovascular protective effect of OT. There are three pathways affecting cardiovascular activity. Neuronal activation of SON and PVN can counteract stress, hypertension, and brain-heart syndrome. Release of OT from MNCs and CMs inhibits atherosclerotic injuries of heart and artery by inhibiting immunological injuries and metabolic disorders through immune protection, paracrine function, and CM regeneration. Finally, activation of OT in pPVN regulates autonomic and vasomotor center activity, leading to inhibition of cardiac arrhythmia, ischemia-reperfusion injury, oxygen demand, and vasoconstriction. Ach, acetylcholine; CM, cardiomyocyte; MNCs, magnocellular neuroendocrine cells; OT, oxytocin; OTR, oxytocin receptor; pPVN, parvocellular division of the PVN; PVN, paraventricular nucleus; SON, supraoptic nucleus.

Oxytocin exerts much protective functions on the CVS through suppressing atherosclerosis-evoking factors and reducing the injury following MI. These protective effects of OT are based on its direct CVS effects and its modulation of the regulatory system of CVS activity (Figure 1).

### Peripheral Effect

In response to environmetal challenges, OT in the hypothalamus can be released into the blood stream in various amounts and patterns (Hatton and Wang, 2008; Hou et al., 2016) and modulate activities of the heart and blood vessels by activating OTRs along with intracardiac OT (Wsol et al., 2016). OT has both chronotropic and inotropic effects on cardiac activity, which can protect the heart from I/R-induced myocardial injury by reducing oxygen consumption. OT also regulates lipid metabolism, and exerts the effect of anti-diabetes, anti-inflammation, and anti-apoptosis while promotes angiogenesis and regeneration of cardiomyocytes as further stated below.

#### Regulation of Lipid Metabolism

Among many factors contributing to atherosclerosis, disorders in the regulation of lipid metabolism and obesity are major etiologies (Schinzari et al., 2017) and thus the target of cardioprotective effect of OT. As reported that hyperlipidemia disrupted OTR signaling (Padol et al., 2017), that serum OT levels were decreased in obese group (Qian et al., 2014), and that mice with OT- or OTR-deficiency developed late-onset obesity (Takayanagi et al., 2008) which was associated with type 2 diabetes and CAD (Amri and Pisani, 2016). Consistently, blood OT concentration was inversely correlated to serum triglyceride, low-density lipoprotein and total cholesterol levels (Qian et al., 2014). It was also found that cholesterol levels in rats displayed a tendency to fall in response to subcutaneous injection of OT (Suva et al., 1980); chronic systemic treatment with OT largely reproduced the effects of central administration of OT by reducing weight gain in obese rodents; chronic subcutaneous or intranasal OT treatment was sufficient to elicit body weight loss in obese subjects (Blevins and Baskin, 2015). Thus, increasing OTR signaling is an important step in prevention of atherosclerosis and OT has therapeutic potential in reducing obesity, atherosclerosis, and the incidence of CAD through regulating lipid metabolism.

#### Anti-diabetic Effects

Population with diabetes mellitus has high prevalence of CAD, peripheral vascular disease and heart failure (Lehrke and Marx, 2017). In type 2 diabetes mellitus, serum OT levels were decreased (Qian et al., 2014). Similarly, in a mouse model of type 2 diabetes mellitus, there was a significant down-regulation of OT, OTRs, ANP, and eNOS gene expressions in the heart, and chronic OT treatment prevented the development of diabetic cardiomyopathy in these animals (Plante et al., 2015). Consistently, OT administered to fasted male subjects via intranasal approach attenuated the peak excursion of plasma glucose (Klement et al., 2017). These findings support that disruption of OTR signaling is closely related to the occurrence of diabetes mellitus.

Further studies revealed that the anti-diabetic effect of OT is closely related to its regulation of glucose metabolism. OT could promote glucose metabolism in cultured cardiomyocytes from newborn and adult rats (Florian et al., 2010), in myocardial cells during hypoxia and other physiological stressors (Shioi et al., 2000) and in mesenchymal stem cells (Noiseux et al., 2012). These findings are consistent with the report that OT stimulated glucose oxidation in myometrial tissue (Okabe et al., 1985) and glucose oxidation and lipogenesis in rat epididymal adipocytes (Goren et al., 1986). This effect was mediated through tricarboxylic acid cycle (Okabe et al., 1985) in an extracellular Ca^2+^-dependent manner (Bonne et al., 1978). On the other hand, OT contributes to metabolic control of glucose by directly improving pancreatic functions. In mice, OTR signaling reduced the death of pancreatic β-cells in islets exposed to cytotoxic stresses, which was diminished in islets isolated from OTR knockout mice (Watanabe et al., 2016). In fasted male subjects, OT was also found to augment the early increases in insulin and C-peptide concentrations in response to glucose challenge due to a pronounced increase in β-cell sensitivity (Klement et al., 2017). These facts indicate that OT exerts anti-diabetic effect by regulating glucose metabolism and protecting pancreatic β-cells in islets, thereby rendering the OT system a potential target of anti-diabetic treatment and prevention of CAD.

#### Anti-inflammation

One of the critical factors involved in the development of CAD is chronic inflammation in association with oxidative stress and the release of pro-inflammatory cytokines (Mullenix et al., 2005). Early atherosclerosis formation is based on leukocyte accumulation and atheroma-activating cells, such as macrophages, dendritic cells, and T- and B-lymphocytes. Pro-inflammatory cytokines enhance leukocyte adhesion molecule expression, leading to leukocyte penetration into the endothelial layer and accumulation in intima. Other molecules, such as interleukin-6, that affect lipid metabolism (Yudkin, 2003) are associated with increased level of C-reactive protein production, which changes inversely to serum OT levels (Qian et al., 2014). OT was also identified as an agent that suppresses the production of inflammatory cytokines (Wang, 2016), including smooth muscle and vascular endothelial cells (Szeto et al., 2013). Moreover, OT could down-regulate neutrophil chemotactic molecules and myocardial neutrophil infiltration, and prevent myocardial injury by reducing inflammatory reaction and reactive oxygen species (ROS) produced by neutrophils (Al-Amran and Shahkolahi, 2013). Thus, along with the general immunological regulatory functions (Wang P. et al., 2015; Wang, 2016), OT could be a potentially preventative agent in those at high-risk for atherosclerosis development and further limit the progression in those with existing disease.

#### Regeneration of Cardiomyocytes

In MI, an initial ischemic event can lead to either reversible or irreversible myocardial injury based on the duration and size of ischemia, and subsequent damages by reperfusion. Prolonged ischemia caused dysfunction in ATPase-dependent ion transport, cellular swelling and rupture, intracellular ion dysregulation and cellular apoptosis; reperfusion caused transient elevation in levels of ROS and inflammatory neutrophils, leading to exacerbation of initial ischemic event; following an ischemic attack, up to 1-billion cardiac cells died but humans had a limited ability to regenerate myocardial cells and injured cells were often replaced by fibrotic scars, leading to conduction abnormalities and heart failure (Rahman and Woollard, 2017; Sanin et al., 2017). For this reason, regeneration of cardiomyocytes is extremely important in long-term prognosis.

Along with the anti-inflammatory and anti-apoptotic effects of OT on the CVS (Kobayashi et al., 2009; Noiseux et al., 2012; Al-Amran and Shahkolahi, 2013), OTR signaling can exert cardioprotective function by promoting regeneration of injured cardiomyocytes. OT stimulated *in situ* differentiation of cardiac stem cells into functionally matured cardiomyocytes by replacing lost cells from ischemic events. When OT-treated mesenchymal stem cells were co-cultured with I/R rat cardiomyocytes, there were decreased cardiac fibrosis, macrophage infiltration, restoration of connexin 43, and increased overall cardiac ejection fraction (Kim et al., 2012). OT preconditioning was also known to increase expression of genes involved in angiogenesis, anti-apoptosis, and anti-cardiac remodeling, such as HSP 27, HSP 32, and VEGF (Gutkowska et al., 2009). Thus, OT treatment can evoke mesenchymal stem cell differentiation to replace the lost cardiac cells, which endows OT the potential of reversing injuries from atherosclerotic CVD.

#### Effects of Intracardiac OT via ANP

In rat heart (Wsol et al., 2016), the right atrium has the highest OT concentration (∼2.128 ng/mg protein) (Jankowski et al., 1998), comparable with OT content in the hypothalamus wherein different regions have OT concentrations varying from >0.1∼228 ng/mg protein (Gainer, 2011). Thus, when OT is released from the atrium, dramatic changes in the cardiac activity can be elicited through paracrine functions, in which ANP serves as cardioprotective mediators of OT in the heart. Upon activation of OTRs, intracellular Ca^2+^ mobilization occurred in the right atrium, which caused ANP release from cardiomyocytes (Gutkowska et al., 2014) whereas, application of OT antagonist blocked basal ANP release (Paquin et al., 2002) and caused a significant decline in ejection fraction and increased cardiac fibrosis (Jankowski et al., 2010b).

Similarly, OTR signaling also increased NO production (Polshekan et al., 2019) that exerted cardiovascular protective effect (Jackson et al., 2017). This action is likely mediated by ANP (Menaouar et al., 2014). Moreover, OT-evoked release of ANP into the blood during expansion of blood volume could also reduce BP through its diuresis and natriuresis effects (Soares et al., 1999). The natriuretic effect helps to remove excess volume and thus reduces BP. These features allow OT to modulate cardiovascular activity through changing ANP secretion.

### Central Effect

Cardiovascular activity is under intense regulation of the central nervous system. CVDs can be caused by disorders in the cardiovascular regulation involving disrupting normal neuroendocrine, autonomic, and behavioral responses. By antagonizing these responses, OT can counteract the deleterious effects of stress, hypertension, unhealthy life-style, and brain-heart syndrome on the CVS.

#### Neural Regulation of Cardiovascular Activity

Vasomotor center(s) in the brainstem are the key structure in neural regulation of cardiovascular activity and are also the target of OT protection of cardiovascular activity. In response to physiological challenges, OT in the hypothalamus can change vasotone through autonomic nerves and in turn modulate cardiovascular activity.

The pumping effectiveness of the heart and contractility of blood vessels are primarily regulated by the autonomic nervous system including excitatory sympathetic and inhibitory parasympathetic nerves. As evidenced in trained rats, there were an increased gain of baroreflex control of heart rate, markedly elevated OT mRNA expression and OT peptide density in PVN neurons, which were blocked with sinoaortic denervation (Cavalleri et al., 2011). OT neurons and their terminals are present in both intra- and extra-hypothalamic sites (Hou et al., 2016), through which OT can change the activity of the neural centers controlling CVS at different levels. The descending fibers from hypothalamic OT cells were found to innervate the locus coeruleus and dorsal vagal complex in the brainstem of the rat (Swanson and Hartman, 1980; Llewellyn-Smith et al., 2012). Through them, OT increased parasympathetic cardiac control and decreased sympathetic cardiac control by activating brainstem vagal neurons (Olszewski et al., 2010), resulting in the slowing down of the heart rate (Higa et al., 2002).

It is worth noting that acute MI could activate microglial P2X7R in the PVN that mediates sympatho-excitatory responses and the production of proinflammatory cytokines in rats. In addition, pro-inflammatory cytokines subsequently increased OT release (Du et al., 2015), thereby limiting the damaging effect of sympathetic outflows (Nielsen et al., 1997; Roy et al., 2018).

#### OT and Hypertension

Hypertension was one of the most significant risk factors for atherosclerosis and the development of CVD, and responsible for 54% of all strokes and 47% of ischemic cardiomyopathy (Lawes et al., 2008). In a cohort study on patients without baseline CVD, about 63% of those with baseline hypertension developed CVD while only 46% in those with normal baseline BP developed CVD (Rapsomaniki et al., 2014).

Oxytocin is deeply involved in body defense against hypertension. As reported, intrauterine growth restriction, caused by excessive glucocorticoid exposure to the fetus, produced hypertension later in life due to damages to OTR signaling (Vargas-Martinez et al., 2017). In hypertensive rats, a decreased expression of OT mRNA and protein was found in hypothalamus. This is consistent with an earlier finding that when OT was injected subcutaneously or intracerebroventricularly for 5 day, BP decreased in rats (Petersson and Uvnas-Moberg, 2008). Moreover, centrally released OT was also found to reduce the cardiovascular responses in BP and heart rate to the acute stressor significantly, which were reversed by OTR antagonist applied through brain ventricular system (Wsol et al., 2009).

The central anti-hypertensive effect of OT is mediated by PVN-brainstem-autonomic nervous system. It has been known for long that OT could reduce overall sympathetic activation of vessel contraction (Olszewski et al., 2010), and selectively dilate blood vessels that were innervated by parasympathetic nerves (Nielsen et al., 1997). Chronic activation of OT neurons restored the release of OT from PVN fibers in the dorsal motor nucleus of the vagus, and prevented the hypertension that occurred with 3 weeks of chronic intermittent hypoxia-hypercapnia exposure (Jameson et al., 2016). Thus, promoting OTergic drive from PVN to brainstem could improve autonomic control of the circulation to maintain stability of the BP.

Lastly, disorder of the immune system in the pathogenesis of hypertension has been firmly established by a large number of investigations (Rodriguez-Iturbe et al., 2017); hence, OT could also reduce hypertension through its immune homeostatic functions (Wang P. et al., 2015; Wang, 2016). Thus, OT can exert anti-hypertensive effects through multiple approaches.

#### Cardiovascular Protection Through Anti-stress Effects

Both positive and negative social stimuli can modify the activity of HPA axis and thus, affect body recovery from acute illnesses including stress, wounds, stroke, and cardiovascular complications (Dupont et al., 2014). OT can exert cardiovascular protection through its anti-stress effects. It has been demonstrated that Watanabe Heritable Hyperlipidemic rabbits exposed to a consistent, stable social experience in association with higher blood OT levels exhibited more affiliative social behavior and less aortic atherosclerosis (Szeto et al., 2013). Social stress promoted the progression of atherosclerosis in these rabbits in association with increased urinary norepinephrine, plasma cortisol and splenic weight as well as less affiliative behavior and more stressful physiological and tissue responses (Noller et al., 2013). In human being, OT was positively associated with diminished stress among securely attached participants and had an attenuating effect on perceived stress due to adverse life events in old age (Emeny et al., 2015). Moreover, in adult mice grouped into isolated or paired environment, social pairing enhanced hypothalamic OT gene expression and that was associated with smaller infarct size, and reduced neuroinflammation and oxidative stress following stroke. In contrast, administration of OT to socially isolated mice reproduced the neuroprotection conferred by social housing, and this effect was associated with the suppressive action of OT on microglia, a source of brain inflammatory cytokines (Karelina et al., 2011). By acting on many brain sites, OT could reduce stress-elicited neuroendocrine, autonomic, and behavioral responses (Li et al., 2017) wherein OT reduced stress-associated release of epinephrine (Wronska et al., 2017), which can reduce cardiac consumption of oxygen and thus endow OT the ability to oppose the injury from stress on the CVS.

#### Suppression of Smoking and Alcohol Craving

Healthy behaviors including moderate alcohol consumption, smoking abstinence, lack of abdominal adiposity, decreased sedentarism, and adherence to Alternate Mediterranean Dietary Index that is characterized by high intakes of fruit, vegetables, fish, and whole grains, moderate amounts of alcohol and dairy products, and low amounts of red or processed meats and sweets, could significantly reduce the presence of coronary artery calcium and plaques in femoral and carotid arteries; adoption of multiple healthy lifestyle behaviors early in life could be a key strategy in tackling the onset of atherosclerosis and reducing the burden of CVD (Dennis and Gerstman, 2014). However, cigarette smoking and alcoholism remain significant problems among population that is at high-risk for atherogenesis.

In a well-characterized, multi-ethnic United States cohort, it has been found that coronary artery calcium was predictive of atherosclerotic CVD in 6.7% of all smokers and in 14.2% of lung cancer screening eligible smokers (Kenkel et al., 2014). Cigarette smoking decreased OT secretion while worsening CAD (Vardavas and Panagiotakos, 2009). Smoking in men inhibited OT release by the mediation of endogenous opioids (Seckl et al., 1988) and GABA (Chiodera et al., 1993). In contrast, nasal application of OT significantly reduced levels of cue-induced smoking craving that often led to smoking relapse (Miller et al., 2016). In addition, OT could decrease withdrawal signs in rats and somatic component of the nicotine withdrawal syndrome (Manbeck et al., 2014). Thus, OT is helpful in reducing these detrimental behaviors.

Similar to the effect on smoking, OT can also inhibit craving for alcohol. The primary metabolite of alcohol, acetaldehyde, stimulated vascular smooth muscle cell Notch signaling and muscle growth, and mediated the ultimate effects of drinking on CVD (Patrick and Ames, 2014). Animal studies support OT as a potential treatment in reducing alcohol consumption. For example, intraperitoneal application of OT (3.0 mg/kg) significantly reduced alcohol (15%) consumption in the first-hour after treatment (Stevenson et al., 2017). Moreover, acute intracerebroventricular infusion of OT attenuated voluntary alcohol self-administration (20%) in male rats. Furthermore, intracerebroventricular application of OT completely blocked alcohol-induced dopamine release within the nucleus accumbens (Peters et al., 2017) that is a well-known nucleus of rewarding. Mechanistically, OT was considered to act by inhibiting the effects of the corticotropin-releasing factor on GABAergic interneurons in the amygdala and PVN, which suppressed the mechanisms of relapse and craving by reducing anxiety, stress vulnerability, and social withdrawal in abstinent alcohol-dependent patients (Faehrmann et al., 2018). By improving these life-styles, OT may also help to suppress the development of atherosclerotic CVD.

#### Protection From Brain-Heart Syndrome

Brain-heart syndrome is reversible acute heart diseases caused by acute encephalopathy involving the regulatory centers of the CVS. Many autonomic brain regions, including insula cortex, amygdala complex, anterior cingulate cortex, ventral medial prefrontal cortex, hypothalamus, and pineal gland are involved in the regulation of cardiovascular activity. At the cellular level, the disturbance of autonomic regulation resulted in catecholamine excitotoxicity, oxidative stress, and free radical myocardium injury (Gotovina et al., 2018). The damage of these structures leads to arrhythmia in previously intact myocardium, systolic and diastolic dysfunction, and ischemic changes. Although it does not cause CAD directly, it often occurs on the basis of atherosclerosis and worsens the CAD.

In association with the stress-relieving effect, OT could also alleviate brain-heart syndrome through its neuroprotective functions. As reported that in a rat model of transient middle cerebral artery occlusion, OT significantly reduced the infarct volume of the cerebral cortex and striatum (Reed et al., 2019). Moreover, intracerebroventricular infusion of OT and centrally released OT induced a preconditioning effect in I/R rat heart via brain receptors (Moghimian et al., 2013). Thus, while OT system dysfunction serves as one common mechanism underlying metabolic syndrome and psychotic disorders (Quintana et al., 2017), brain OT can exert cardiovascular protective effect by suppressing the brain-heart syndrome in cerebrovascular accidents.

## Signaling Pathways Mediating the Cardiovascular Protective Effect of OT

Oxytocin is known to exert its biological functions through both OTR and VP receptors (Song and Albers, 2017). Although supraphysiological doses of OT could also activate VP receptors, OTR is the mediator of OT effects in terms of cardiovascular protection (Wsol et al., 2014). Understanding of the signaling pathway through OTR signaling is crucial in understanding of its cardiovascular protection and identifying novel targets of treatment.

### OTR and G Proteins

Oxytocin receptors are typical class I G protein-coupled receptors (GPCRs). The binding of OT to OTRs activates its primary downstream effector Gq protein via RVSSVKL segment in the COOH-terminal region of the third intracellular domain of OTR (Zhong et al., 2007); however, Gi/o family members can also mediate the cardioprotective effects of OT directly as stated below.

In general, the effect of OTR activation is mediated by phospholipase C (PLC)-β downstream to the α-subunit of both Gq and Gi/o proteins (Busnelli et al., 2013; Jurek and Neumann, 2018). In the early stage of cardiac injury, the activity of PLC-β1b was elevated selectively, which caused de-phosphorylation of phospholamban and depletion of the Ca^2+^ stores in the sarcoplasmic reticulum (SR), leading to cytosolic Ca^2+^ oscillation, mitochondrial Ca^2+^overload, and oxidative stress (Abdallah et al., 2011). Thus, it is not likely for OTR to activate PLC-β1 signaling to exert the cardioprotective function. Instead, PLC-β3 could be a mediator of OTR signaling (Yue and Sanborn, 2001). The differential effects of downstream signals to OTR are in agreement with the findings that biological effects of OT depended on OTR localization in caveolin-1 enriched domains (Rimoldi et al., 2003) and that angiotensin and OT respectively caused injury and protective effects by activating different signaling pathways downstream to their corresponding Gq proteins (Natochin et al., 2018).

The activation of OTRs can also protect cardiomyocytes through Gβγ subunits of OTR-coupled G proteins and the crosstalk between this GPCR and receptor tyrosine kinase signaling pathways, which has been identified in myometrial cells (Zhong et al., 2003) and HEK293 cells (Rimoldi et al., 2003) wherein Gβγ subunits could increase phosphorylation of extracellular signal-regulated protein kinase (ERK) 1/2, the critical component of cardioprotective reperfusion injury salvage kinase (RISK) pathway (Polshekan et al., 2016). The RISK pathway involves phosphatidylinositol 3-kinase (PI3K)-protein kinase B (Akt)-eNOS cascades and ERK 1/2. In addition, AMP-activated protein kinase (AMPK), Ca^2+^/calmodulin-dependent protein kinase (CaMK) signaling and others are also implicated, disruption of which has been implicated in immunometabolic dysregulation-associated pathogenesis of cardiac arrhythmias as recently reviewed (Wang S.C. et al., 2018). The activation of OTR-Gq and Gi/o proteins could regulate these and other signaling pathways to suppress immunometabolic dysregulation-associated CAD.

Between the signaling events downstream to α-subunit and Gβγ subunits of OTR-coupled G proteins, there is also crosstalk. Indeed, the activation of PLC could be generated by the βγ complexes released by Gαi of the OTR/Gi coupled receptor and by transactivating tyrosine kinase receptor EGFR via MAPK cascade (Rimoldi et al., 2003).

It is important to note that there are rapid and extensive internalization and desensitization of the OTR upon agonist exposure, which is determined by several signaling molecules in a cascade. For example, stable OTR/beta-arrestin2 interaction played an important role in determining the rate of recycling of human OTRs; OTRs were localized in vesicles containing Rab5 and Rab4 small GTPases, the markers for direct receptor recycling without decomposition (Conti et al., 2009). In human embryonic kidney cells, OTR internalization was unaffected by inhibitors of protein kinase C (PKC) or CaMK-II but was significantly reduced after transfection with dominant-negative mutant cDNAs of GPCR kinase (GRK)2, β-arrestin 2, dynamin, and Eps15 (a component of clathrin-coated pits) (Patel and Radeos, 2018); GRK-evoked OTR phosphorylation was a prerequisite for β-arrestin-mediated internalization and OTR desensitization (Wang C. et al., 2018). In uterus, knockdown of GRK6 largely prevented OT-induced OTR desensitization; in contrast, selective depletion of GRKs 2, 3, or 5 was without effect (Denson et al., 2018; Liu et al., 2018). This signaling feature highlights a potentially beneficial effect of using intermittent OT application pattern in treating CVDs, the power of which has been well-discussed about studies on the milk-letdown reflex (Hatton and Wang, 2008; Hou et al., 2016).

### PI3K/Akt Cascades

Disruption of PI3K/Akt cascade is a major pathological event for CAD occurrence, which has been observed in smoking interference of the cardioprotective signaling by post-conditioning (Guasch and Gilsanz, 2016) and many other etiologies as recently reviewed (Wang S.C. et al., 2018). The protective effect of this pathway is supported by the findings of myocardial protection through hydrogen sulfate (H_2_S) (Jin et al., 2017), isoflurane-induced myocardial post-conditioning under acute hyperglycemia (Raphael et al., 2015), the protection of the heart against I/R injury by limb remote ischemia preconditioning through the opioid system (Zhang et al., 2017), post-reperfusion administration of granulocyte colony-stimulating factor (Sumi et al., 2010) and others (Zhou et al., 2015; Zafirovic et al., 2017). The protective effect of this pathway is likely associated with its critical roles in cellular proliferation, migration, and protection against apoptotic and cytotoxic effects due to hypoxia (Wang S.C. et al., 2018). Consistently, PI3K/Akt cascade is also implicated in the cardioprotective effect of OT. For example, activating PI3K/Akt signaling was responsible for the post-conditioning and the anti-apoptotic effect of OT (Gonzalez-Reyes et al., 2015) whereas, PI3K/Akt inhibitors and OTR blocker atosiban blocked the protective effect in rats (Polshekan et al., 2016).

Oxytocin activation of PI3K/Akt signaling could be achieved through a crosstalk between Gq protein and epidermal growth factor receptor (Zhong et al., 2003) that is an upstream signal of PI3K/Akt as demonstrated in the action of protease-activated receptor 2 (Wang and DeFea, 2006; Wang et al., 2010). In OTR signaling, PI3K/Akt signaling-mediated protection is achieved through eNOS that subsequently activates mitochondrial ATP-dependent potassium (mKATP) channels (Das and Sarkar, 2012) to reduce mitochondrial oxidative stress (MOS) as discussed in previous review in detail (Wang S.C. et al., 2018).

Between NO and mKATP channels, the involvement of NO-soluble guanylyl cyclase has been found in OT-evoked differentiation of porcine bone marrow stem cells into cardiomyocytes and cell proliferation (Ybarra et al., 2011). In cells treated with OT, activated Akt and eNOS were translocated into the nuclear and perinuclear area to protect heart from I/R injury, which was abrogated by inhibition of OTR signaling, PI3K, cGMP-dependent protein kinase as well as soluble guanylate cyclase (Gonzalez-Reyes et al., 2015). Downstream to the eNOS-cGMP-dependent protein kinase is the mKATP channels, inhibition of which is a pivotal mechanism in immunometabolic disorder-evoked CVDs (Wang S.C. et al., 2018). In addition, cyclic AMP response element-binding protein (CREB) signaling could also be a mediator of PI3K/Akt signaling, which has been shown in the nervous system (Da Silva et al., 2013).

### ERK 1/2 Pathway

Following the activation of OTRs, the release of Gβγ subunits from OTR-associated Gq protein can activate ERK 1/2 as shown in myometrial cells (Zhong et al., 2003) and in OT neurons (Wang and Hatton, 2007a,b). As a major component of the RISK pathway, ERK 1/2 signaling is also implicated in OT-mediated cardiovascular protection. For example, the cardioprotective effect of OT post-conditioning on isolated ischemic rat heart depended on the activation of ERK1/2 signaling (Gonzalez-Reyes et al., 2015) since that was blocked by ERK1/2 inhibitors and atosiban (Polshekan et al., 2016).

Studies further revealed that the protective effect of ERK1/2 signaling is mediated through CREB signaling. Reduced expression of genes regulated by the transcription factor CREB is linked to atrial fibrillation susceptibility in patients, which has been verified in transgenic mouse model recently (Seidl et al., 2017). CREB is responsible for the expression of potassium channel Kv1.5 that was impaired in diet-induced obese in mice (Huang et al., 2013). Thus, this signaling cascade is critical in pathophysiology of atrial fibrillation, ventricular ectopy, insulin secretion, hypoxic pulmonary vasoconstriction and sudden cardiac death. Moreover, CREB is also associated with peroxisome proliferator-activated receptor signaling known to regulate lipid metabolism and insulin sensitivity, integrity of sarcomeres and mitochondria, and the deposition of collagen and glycogen in the heart (Seidl et al., 2017). In addition, GRK2 may participate in OT regulation of CVS activity although the expression level of GRK2 was dependent on the tissues and their functional status (Montgomery et al., 2018). Importantly, high fat-diet caused marked intracellular lipid accumulation and significantly increased cardiac GRK2 levels in mice, which promoted obesity-induced cardiac remodeling and steatosis. In contrast, low GRK2 protein levels were able to keep the PKA/CREB pathway active and prevented a high fat diet-induced down-regulation of key fatty acid metabolism modulators such as peroxisome proliferator-activated receptor gamma co-activators, thus preserving the expression of cardioprotective proteins such as mitochondrial fusion markers mitofusin (Marir et al., 2013).

Mitofusin was known as an inhibitor of mitochondrial membrane depolarization and ROS production by acting on the mitochondria-associated ER membrane (MAM) to inhibit mitochondrial Ca^2+^ overloading, a function opposite to glycogen synthase kinase-3β (GSK-3β) (Jankowski et al., 2010b). Thus, by activating CREB signaling, OT-activated ERK 1/2 can suppress ER stress response (Watanabe et al., 2016) and the associated MOS (Wang S.C. et al., 2018), thereby exerting cardiovascular protective effects. Interestingly, the protective effect of CREB signaling might not work in low potassium diet since elevated autophagy and CREB signaling were found to promote calcification of arteries from low potassium diet-fed mice as well as aortic arteries exposed to low potassium *ex vivo* (Brown et al., 2013).

### CaMK Signaling

Another important signaling molecule in OT protective effect is CaMK-II. CaMK II is important for Ca^2+^ homeostasis of cardiomyocytes. In infarcted heart, cardiac SR Ca^2+^ uptake and release activities were depressed significantly due to a decrease in SR CaMK-II phosphorylation of the SR proteins, ryanodine receptor, Ca^2+^ pump ATPase/ER Ca^2+^ ATPase, and phospholamban (Araujo et al., 2013), leading to ER stress and MOS. Thus, CaMK could be an important mediator of the cardiovascular protection of OT.

Following the activation of OTRs, mobilization of Gαq or Gβγ caused intracellular Ca^2+^ release and subsequent activation of CaMK-II (Yue and Sanborn, 2001), likely mediated by PLC-β3 signaling pathway. The intracellular Ca^2+^ could also come from other sources. For instance, L-type Ca^2+^ channels, IP3-RyR-gated, and store-operated Ca^2+^ channels including transient receptor potential channel pathways played significant roles in OT-induced contractions of myometrium of buffaloes (Borrow et al., 2018). CaMK-II can further phosphorylate PLC-β3 but not PLC-β1 (Yue and Sanborn, 2001) that was a known as a deteriorating signal in heart (Abdallah et al., 2011). The activation of CaMK-II was associated with OT-elicited activation of AMPK (Lee et al., 2008), release of ANP (Gutkowska et al., 2014) and NO (Lee et al., 2008; Menaouar et al., 2014), and they are all known to play important roles in OT protection of CVS.

It has been demonstrated that OT could antagonize endothelin-1 or angiotensin II-evoked cardiomyocyte hypertrophy though ANP and NO release in the developing rat heart, which was mediated by CaMK-II and AMPK pathways and by normalization of the reduced Akt phosphorylation (Menaouar et al., 2014). Moreover, CaMK II was involved in NO-elicited relaxation of endothelium-intact rat aortic rings as a result of Ca^2+^-dependent activation of eNOS in cultured porcine aortic endothelial cells (Alizadeh and Mirzabeglo, 2013). Thus, CaMK signaling is an important approach for OT protection of arteries.

### AMPK Pathway

In parallel with the RISK pathway, AMPK signaling pathway is also involved in OT function in anti-inflammation and promotion of metabolic homeostasis (Mancini et al., 2017) through multiple approaches. As reported that OT stimulated and activated AMPK in C2C12 myoblast cells in a time/dose-dependent manner. This process also depends on the activation of CaMK since it was blocked by inhibition of either CaMK or AMPK (Lee et al., 2008). In db/db mice, OT treatment normalized cardiac structure and function, cardiac OTRs, ANP, and AMPK while reducing body fat accumulation, fasting blood glucose levels and improving glucose tolerance and insulin sensitivity (Plante et al., 2015). Moreover, AMPK could inhibit HMG-CoA reductase to reduce cholesterol synthesis and inflammation (Vilahur et al., 2014). Thus, inhibition of multiple pro-inflammatory signaling pathways and metabolic disorders by AMPK should be an important mechanism of the cardiovascular protective functions by OT.

### Other Signals

Oxytocin protection of the CVS was associated with its activation of PKC (Faghihi et al., 2012), likely PKC-ε signaling in the mitochondria. In cardioprotection, PKC-ε, a downstream effector of PLC-β and NO generated by eNOS, increased the stability of gap junctions and suppressed ventricular fibrillation by antioxidant-increased connexin-43 in rats (Bacova et al., 2017; Lee et al., 2017). In contrast, activation of PKC-α, a downstream event of PLC-β1b that is a heart-specific signaling, led to cardiac injury by increasing inducible NOS (iNOS) expression, concomitant to enhanced apoptotic cell percentage, and molecular interaction between apoptotic protease activating factor-1 and cytochrome C (Qiu et al., 2012). These findings indicate that PLC-β1b-PKC-α-iNOS signaling pathway activates mitochondrial apoptotic pathways while PKC-ε is protective. In contrast to the effect of OT, norepinephrine, angiotensin II, and endothelin 1 and phorbol ester could activate and translocate protein kinase D1 to the Z-disks in rat cardiomyocytes in a PKC-ε-dependent manner, which process was essential to induce hypertrophic responses (Iwata et al., 2005). Thus, protein kinase D1 and PKC-ε interaction may also induce cardiac hypertrophy. Moreover, ERK1/2 activation by metabotropic glutamate receptor 1 induced melanoma development and was also PKC-ε-dependent, but cAMP and PKA-independent. Thus, the proliferating effect of PKC-ε downstream to OTRs either functions during regeneration of injured cardiomyocytes or is linked to different signaling events from those that are used by the GPCRs of “stress hormones.” Nevertheless, experimental evidence remains to be collected.

In parallel with NO, H_2_S is also a well-known cardioprotective gaseous signal (Raphael et al., 2015; Jin et al., 2017). H_2_S is mainly converted from cysteine catalyzed by cystathionine-γ-lyase (CSE) that was present in OTR-expressing supraoptic neurons (Coletti et al., 2019) and could be activated by CaMK, PI3K and NO. H_2_S could also suppress inflammation by activating KATP channel, PI3K, and pERK1/2 signaling (You et al., 2017). Importantly, CSE can regulate OTR expression in tissue- and function-dependent manner. In isolated human uterine smooth muscle cells, CSE had negative correlation with the expression of OTR in pregnant myometrial tissues (You et al., 2017). By contrast, myocardial injury evoked by contusion of the thorax in mice was found to reduce myocardial OTR expression, and that was aggravated in CSE(-/-) mice; exogenous H_2_S administration restored myocardial OTR protein expression to wild-type levels (Merz et al., 2018). This study suggests that cardiac CSE can exert cardioprotective function by activating RISK pathway and up-regulating cardiac OTR expression.

In addition, OT-evoked protection was also related to increases in VEGF, B-cell lymphoma 2 and matrix metalloproteinase-1 (Kobayashi et al., 2009) along with aforementioned signals that opened mKATP channels (Alizadeh et al., 2010). Among them, serum levels of VEGF had negative association with atrial fibrillation episode duration (Peller et al., 2017) and exerted antifibrotic and angiogenic effects, which were associated with the activation of matrix metalloproteinase-1 and eNOS (Kobayashi et al., 2009).

### Signaling Network

In cardiovascular protection, different signaling pathways function interactively and coordinately to suppress immunometabolic disorders. For instance, exercise in mice reduced infarct size by 60% while increasing phosphorylation of Akt, ERK1/2, and AMPK; however, the level of corresponding phosphatases PTEN, MKP-3, and PP2C were decreased in both wild-type and obese mice (Danalache et al., 2014). Moreover, different signaling pathways have close interactions as shown in the following studies. In vascular endothelial cells in mice (Chen et al., 2009) and human umbilical vein endothelial cells (Huang et al., 2017), AMPK served as an upstream enzyme of the Akt-NO pathway. These signals are known to activate mKATP channels via activation of eNOS-NO-protein kinase G pathway and CREB, thereby protecting the CVS from the damaging effect of ER stress and MOS as previously discussed (Wang S.C. et al., 2018). Another example is that H_2_S-evoked activation of ERK 1/2 PI3K depended on mKATP channel activation (You et al., 2017).

**FIGURE 2:**
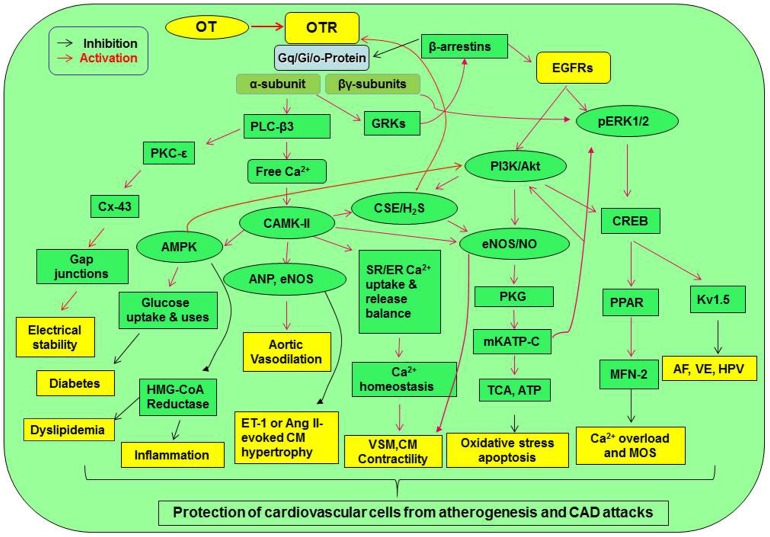
Signaling pathway mediating cardiovascular protection of OT. Activation of OTR-Gq and Gi/o proteins can directly increase Gqα subunit and Gβγ subunit signaling. These signals can cross-activate EGFR while activating their downstream signaling molecules, resulting in the activation of CaMK-AMPK, PKC-ε, PI3K/Akt-eNOS, H_2_S, and ERK1/2-CREB signaling cascades. These signaling cascades lead to the activation of a variety of cardioprotective functions including electrical stability, metabolic arrangement, inhibition of inflammation or oxidative stress, and others. AF, atrial fibrillation; Akt, protein kinase B; AMPK, AMP-activated protein kinase; Ang, angiotensin; CaMK, Ca^2+^/calmodulin-dependent protein kinase; CREB, cyclic AMP response element-binding protein; CSE, cystathionine-γ-lyase; Cx-43, connexin-43; EGFR, epidermal growth factor receptor; eNOS, endothelial nitric oxide synthase; ER, endoplasmic reticulum; ERK1/2, extracellular signal-regulated protein kinase 1/2; ET-1, endothelin-1; GRK, G protein-coupled receptor kinase; HMG-CoA reductase, 3-hydroxy-3-methyl-glutaryl-coenzyme A reductase; HPV, hypoxic pulmonary vasoconstriction; H_2_S, hydrogen sulfate; Kv1.5, voltage-gated potassium channel Kv1.5; MFN-2, mitofusin-2; mKATP C, mitochondrial ATP-dependent potassium channels; MOS, mitochondrial oxidative stress; PI3K, phosphatidylinositol 3-kinases; PKC, protein kinase C; PKG, protein kinase G; PLC-β3, phospholipase C-β3; PPAR, peroxisome proliferator-activated receptor; SR, sarcoplasmic reticulum; TCA cycle, tricarboxylic acid cycle; VSM, vascular smooth muscle; VE, ventricular ectopy. Other annotations refer to Figure 1.

Together with other evidence, such as the mediation of statin protection by the phosphorylated Akt, GSK-3β (inhibition), and CREB and the functions of OT at other tissues (Klein et al., 2014; Watanabe et al., 2016), we propose the presence of an OTR signaling network that protects the CVS from atherosclerotic injury and CAD (Figure 2).

## Protection by Alleviating ER Stress and MOS

Coronary atherosclerosis and CAD are largely caused by immunometabolic disorders and the resultant ER stress and MOS (Wang S.C. et al., 2018). In agreement with the common machinery of immunometabolic regulation, the cardioprotective effect of OT is also dependent on its suppression of immunometabolic disorders involving “ER-mitochondrial syncytium.”

### Reducing ER Stress

The ER is an essential organelle for protein synthesis, folding, translocation, calcium homoeostasis, and lipid biosynthesis. Stimuli that disrupt ER homoeostasis and functions can cause the accumulation of misfolded and unfolded proteins that disrupt ER membrane structure and functions. As an adaptive strategy to restore ER homoeostasis, an unfolded protein response (UPR) occurs following ER stress through activating transcriptional and translational pathways. Maladapted ER stress could worsen ER functions, trigger inflammatory reaction and damage membrane structure, leading to cell dysfunction and apoptosis (Wang et al., 2017). In the CVS, ER stress causes the development and progression of various CVDs. Suppression of ER stress has been shown to promote angiogenesis (Pohl et al., 2018), reduce cardiomyocyte apoptosis, improve heart function in diabetic rats (Rabow et al., 2018) and decrease cell death in ER stress models of cultured neonatal rat cardiomyocytes and in acute MI in mice (Ding et al., 2019). In OT-elicited cardiovascular protection, suppression of ER stress is also an important mechanism.

It has been reported that OT in the colostrum attenuated the impact of inflammation on postnatal gut villi and enhanced autophagy to protect against amino acid insufficiency-induced ER stress during the interval between birth and the first feeding (Klein et al., 2017). OT reduced ER stress by reducing the burden of protein synthesis and processes in the ER through rapamycin complex 1 (mTORC1) signaling that was up-regulated in CVDs (Yano et al., 2016). In gut cells, OT was found to downregulate anabolic effects induced by fresh growth medium catalyzed by mTORC1 through regulation of PI3K/Akt/mTORC1 pathway, which has been identified in mice with malignant arrhythmias, heart failure, and premature death (Cao et al., 2013). Consistently, through activating inositol requiring enzyme (IRE), OT increased the UPR and a chaperone protein, immunoglobulin binding protein while decreased translation initiation factors (Klein et al., 2017). Through this chaperone approach, OT could inhibit lipopolysaccharide-evoked ER stress (Klein et al., 2017). Mechanistic studies revealed that the enhancement of IRE1α (IRE1α)/X box-binding protein-1 (XBP1) activity in turn increased ER-associated degradation-mediated clearance of misfolded proteins and autophagy (Ding et al., 2019). Another line of evidence showed that following the activation of OTRs, IRE1α activation was mediated by OT-elicited VEGF release, which together with sliced XBP1 could carry out the protective functions of OT in a PI3K/Akt/GSK-3β/β-catenin/E2F2-dependent manner (Pohl et al., 2018). Thus, OT can reduce translation of proteins, increase their export and clearance of misfolded proteins during ER stress and thus protect cardiomyocytes from injuries of immunometabolic stress.

### Inhibition of the MOS

The mKATP channel is a key carrier in the cardioprotective effect of OT and a downstream signal in the RISK pathway (Raphael et al., 2015; Jin et al., 2017; You et al., 2017) and AMPK signaling (Chen et al., 2009; Huang et al., 2017). In OT-stimulated cells, activated Akt accumulated intracellularly close to mitochondria in mesenchymal stem cells that have therapeutic potential in I/R heart, and allowed NO-dependent activation of protein kinase G to open mKATP channels (Faghihi et al., 2012). Moreover, OT could activate mKATP channels in the heart of anesthetized rats that were subject to I/R injury and thus significantly decreased infarct size, creatine kinase-MB isoenzyme plasma level, severity and incidence of ventricular arrhythmia. These effects were blocked by atosiban (Alizadeh et al., 2010). These findings are consistent with the cardioprotective effect of activating mKATP channels under other conditions (Das and Sarkar, 2012).

What needs to be note is that the protective effect of OT in mitochondria is achieved in an “oxidative preconditioning” manner. In a simulated I/R experiment using heart-derived H9c2 cells, OT was shown to trigger a short-lived burst in ROS production but reduced I/R-evoked remarkable ROS production (Gonzalez-Reyes et al., 2015). This “oxidative preconditioning” blocks I/R-evoked MOS, thereby exerting the protective effect. By suppressing the MOS, OT also restores tricarboxylic acid cycle and normal ATP production that are critical in cardiac protection (Wang S.C. et al., 2018).

### OT Suppression of Ca^2+^ Overload Through SR/ER-Mitochondrial Network

It is of interest to note that ER stress and MOS do not occur independently in CAD-associated immunometabolic disorders. The two organelles are interconnected through MAM in the heart, which was responsible for Ca^2+^ signaling between ER and mitochondria following the activation of inositol 1,4,5-trisphosphate receptor (IP_3_R) (Wu et al., 2017), thereby forming an ER-mitochondrial channel.

This Ca^2+^ signaling in the heart is regulated by GSK-3β protein in the ER. Dephosphorylation/activation of GSK-3β occurs following the activation of JNK which could be induced by advanced glycation end-products in diverse pathological settings including diabetes, inflammation and acute I/R injury in the heart (Wang S.C. et al., 2018). During I/R, increased GSK-3β activity leads to enhanced transfer of Ca^2+^ from ER to mitochondria by interacting with the IP_3_R Ca^2+^ channeling complex in MAM, leading to cytosolic and mitochondrial Ca^2+^ overload and the resultant cell death. Inhibition of GSK-3β reduced both IP_3_R phosphorylation and ER Ca^2+^ release, which consequently diminished both cytosolic and mitochondrial Ca^2+^concentrations as well as mitochondrial sensitivity to apoptosis (Gomez et al., 2016). Activation of the mKATP channels reversely induced inhibitory phosphorylation of GSK-3β and suppressed substantial ROS production, lactate dehydrogenase release and apoptosis after antimycin washout (Sunaga et al., 2014). Another key molecule regulating the interaction between mitochondria and ER is mitofusin-2. Mitofusin-2 has been identified to suppress the interaction between the ER and mitochondrial apoptotic pathway (Guan et al., 2016; Yang F. et al., 2017), a function opposite to GSK-β.

In addition to the ER, SR is also an important source of mitochondrial Ca^2+^ overload in myocardiac pathogenesis. Ca^2+^ transient from SR could also contribute to the MOS. For example, fructose-rich diet induced decrease in SR-mitochondrial distance, SR Ca^2+^ leak, and Ca^2+^ transit between the two organelles, which resulted in mitochondrial membrane depolarization and oxidative stress, thereby activating the apoptotic pathway and diabetic heart injury (Federico et al., 2017).

**FIGURE 3:**
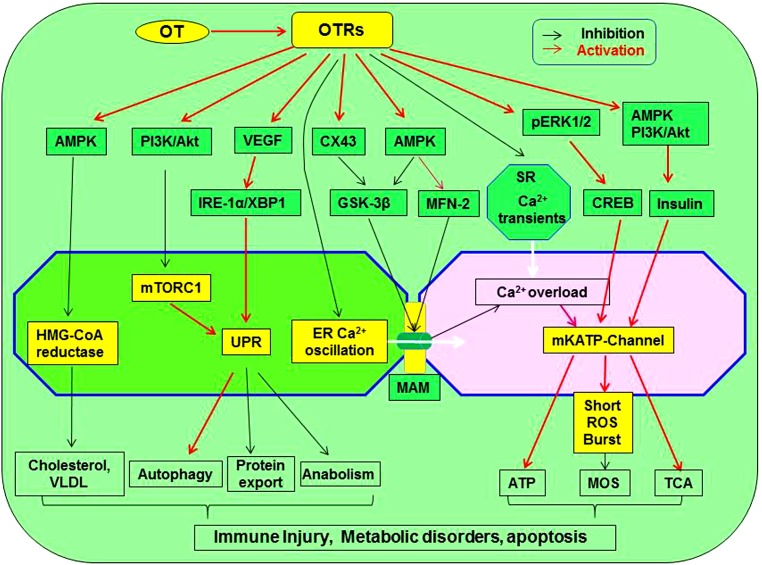
Working model of OT suppression of the malfunctioned ER-mitochondrial communication. OTR activation can inhibit inflammation, ER stress, restore UPR and chaperone functions, reduce the production of cholesterol and VLDL, inhibit ER-mitochondrial Ca^2+^ oscillation and overload through MAM, precondition MOS, restore TCA cycle, and suppress inflammatory and apoptotic pathways, leading to regeneration and repair of injured cardiomyocytes. These processes are signaling through activating AMPK, pERK1/2, PI3K/Akt, VEGF, and CX43 while inhibiting mTORC1, HMG-CoA reductase, GSK-3β, and calcium oscillation. As a result, lipotoxicity and formation of atherosclerosis are inhibited. GSK-3β, glycogen synthase kinase-3β; IRE1α, inositol-requiring enzyme 1α; pERK1/2, extracellular signal-regulated protein kinase 1/2; MAM, mitochondria-associated ER membrane; ROS, reactive oxygen species; mTORC1, rapamycin complex 1; UPR, unfolded protein response; VEGF, vascular endothelial growth factor; VLDL, low-density lipoproteins; XBP1, X box-binding protein-1. Other annotations refer to Figure 2.

Oxytocin could protect the heart by blocking the activity of malfunctioned ER-mitochondrial syncytium through the following approaches. (1) OT can increase cardiac expression of connexin 43 (Gassanov et al., 2008; Kim et al., 2012), which was known to inhibit GSK-3β signaling in cardiomyocytes (Ishikawa et al., 2012). (2) OT can activate PI3K/Akt pathway (Gonzalez-Reyes et al., 2015) that is known to exert antiapoptosis in association with upregulation of mitofusin 2 (Zhang et al., 2014). (3) By activating AMPK, OT can decrease the Ca^2+^ oscillation through increasing mitofusin 2 expression (Wang F. et al., 2015) and suppressing GSK-3β by activation of insulin receptor (Chopra et al., 2012) that was known to increase mitofusin 2 and decrease GSK-3β (Litwiniuk et al., 2016). This possibility is supported by the fact that OT stimulated PKC activity in adipocyte plasma membranes, an effect similar to that of insulin (Egan et al., 1990); however, direct evidence remains to be collected. Figure 3 presents a working model of OT suppression of the malfunctioned ER-mitochondrial communication.

## Limitation for Therapeutic Use of OT

Oxytocin was generally considered a safe agent in clinical usage (Alfirevic et al., 2016). A daily treatment with 40 IU intranasal OT for 4 months did not impact on OT and VP plasma levels nor on cardiovascular, body fluids and food intake parameters in healthy humans (Busnelli et al., 2015). Similarly, intranasal OT strengthened the bonding between male resident and its female partner in rats without changing cardiovascular activity (Calcagnoli et al., 2015). Moreover, OT has the beneficial effects of insulin, antioxidants, and corticosteroids but not their side effects (Hou et al., 2016). Thus, its clinical trial is tempting.

It is important to note that some animal studies administer supraphysiological levels of OT (e.g., micromolar concentrations rather than picomolar levels) and at that dosage, OT has affinity for VP receptors and VP-like effect, such as pressor effect of OT at its initial actions (Petty et al., 1985). Moreover, cardiovascular side effects and allergy to OT have been found in gynecological practice although quite rare. Thus, it is necessary to address these negative sides in clinical trials of OT in prevention and treatment of the CVD.

### Exogenous OT Application

Since chronic CVDs are associated with declines in OT/OTR signaling, restoring OTR signaling with OT becomes a natural selection. To simulate physiological and natural labor, pulsatile application of intravenous OT was commonly used (Saccone et al., 2017). Pulsatile application was associated with lower total dose of OT and less incidence of tachy-systole; however, cardiovascular disturbances like hypotension and reflexive tachycardia were sometimes observed when 5∼10 IU OT was given as a rapid intravenous bolus (Lin et al., 2007; Saccone et al., 2017). In pregnant women with MI, coronary spasm or thrombosis could occur in response to the intravenous bolus of OT. By contrast, there were only modest cardiovascular disturbances during slow infusion (Svanstrom et al., 2008). This fact highlights the necessity to reconsider the optimal drug, dose, and administration route in clinical trials of OT.

It is known that the beneficial effect of OT on cardioprotection is correlated with the basal levels of endogenous OT (Hirst et al., 1991). OT administration to individuals with a low pretreatment OT levels could be beneficial whereas, individuals with an elevated basal OT levels would be prone to adverse effects, which has been shown in swine (Jacquenod et al., 2015). It is likely that during parturition the basal OT levels are already very high, a bolus injection of OT in large amount may reversely decrease OTR signaling by reducing OTR protein expression (Authier et al., 2010). Moreover, high doses of OT also activated VP receptors (Wang and Hatton, 2006), thereby evoking MI (Ying et al., 2015). These facts highlight the necessity to assay the basal levels of blood OT in the induction of labor, particularly in those with CAD, and use drugs like prostaglandins (Mahomed et al., 2018) and misoprostol (Pimentel et al., 2018) to replace OT in those who have higher basal OT levels.

In using exogenous OT to treat CAD, pre-existing neuroendocrine conditions of the patients should also be considered. It has been observed that chronic application of OT and angiotensin-II together increased mean arterial pressure, and caused left ventricular hypertrophy and renal damage in male rats (Phie et al., 2015). It is likely that prolonged administration of OT in CAD patients with elevated basal angiotensin-II levels accelerated angiotensin-II-induced hypertension and renal damage (Gu et al., 2016). Since elevated basal levels of angiotensin-II is common among patients with CAD complications (Schuh et al., 2017), alternative approaches of OT application should be considered.

In addition, anaphylaxis to OT was occasionally observed in delivering women with latex allergy and bronchial asthma (Liccardi et al., 2013). Thus, special attention in exogenous OT application should be paid to patients who have the history of latex allergy as well as history of hypotension, reflexive tachycardia, and high angiotensin levels in delivering women.

### Intranasal OT Delivery

Circulating OT could modulate cardiovascular activity directly (Alfirevic et al., 2016); however, intravenous application of OT is inaccessible to the brain sites that are involved in neural regulation of CVS activity. Thus, intranasal OT application had been tested in heart rate variability-an index of autonomic cardiac control (Quintana et al., 2013). Although intranasally-applied OT usually does not evoke significant change in plasma OT levels (Leng and Ludwig, 2016), it could exert antiarrhythmic effect in human being as reported (Jain et al., 2017; Sack et al., 2017).

Intranasal administration of OT could regulate brain activities including hypothalamic sites (Delorme and Garabedian, 2018) without side effects of peripheral exposure (Busnelli et al., 2015; Calcagnoli et al., 2015). Intranasal delivery of OT had been considered in relieving brain-associated etiologies of CAD, such as obesity, Alzheimer’s disease, depression, anxiety, seizure, and stroke (Chapman et al., 2013). In CAD patients, intranasal OT also exerted the protective effect (Zhang et al., 2013) by suppressing the activity of HPA axis and adrenaline secretion (Yee et al., 2016) and reducing sympathetic output (Tracy et al., 2018). However, intranasal OT may act on multiple brain sites though different nose-brain routes (Veening and Olivier, 2013) that may impose additional complications, such as co-activation of VP neurons, thereby compromising the protective effect of OT (Bartekova et al., 2015). In addition, responsive activation of parvocellular OT neurons during MI could drive cardiac sympathetic nerve activation as observed in rats (Roy et al., 2018), which could worsen the decompensated heart functions. Although it remains to study if intranasal OT application could activate this sympathetic pathway, caution should be taken in using this approach to deliver OT in acute phase of MI. As a whole, how to let OT activate the descending vagal pathway to minimize cardiac injury remains a puzzle in exploring the therapeutic potential of intranasal OT.

## Summary

In varieties of etiologies of atherosclerosis and the resultant CAD, deficits in OTR signaling are an important one. Although the presence of some rare side effects and optimal approaches of OT application remain to be clarified, the perspective to reduce the morbidity and mortality of atherosclerosis and CAD by targeting OTR signaling is highly desirable, which can at least avoid the compromising effect of VP receptor signaling while efficiently blocking the key pathological link in CAD development. To exert the therapeutic potential of OT, questions remain to be answered include but not limit to understandings of the signaling processes from OTR activation to its downstream signals, including CaMK, AMPK, PI3K/Akt, pERK 1/2, PKC-ε, NO and H_2_S, and the details of inter-organelle Ca^2+^transfer and its regulation, and so on. With careful monitoring of both the positive and negative effects of OT, particularly in delivering women (Weissman et al., 2017), future clinical trials of OT therapies would contribute significantly to the translational study in curbing the development of atherosclerosis and the CAD complications.

## Author Contributions

PW and SW wrote the first draft. HZ and Y-FW conceived the study. Y-FW edited the draft. All authors made critical discussion.

## Conflict of Interest Statement

The authors declare that the research was conducted in the absence of any commercial or financial relationships that could be construed as a potential conflict of interest.
